# Retraction Note: ZKSCAN3 drives tumor metastasis via integrin β4/FAK/AKT mediated epithelial-mesenchymal transition in hepatocellular carcinoma

**DOI:** 10.1186/s12935-024-03566-0

**Published:** 2024-11-11

**Authors:** Jieqiong Li, Nan Hao, Juan Han, Mi Zhang, Xiaomei Li, Nan Yang

**Affiliations:** 1 Department of Nurse, The First Affiliated Hospital of Xi’an Jiaotong University, Xi’an, 710061 Shaanxi China; 2 Department of Surgical Oncology, The First Affiliated Hospital of Xi’an Jiaotong University, Xi’an, 710061 Shaanxi China; 3 Department of Intensive Care Unit, The First Affiliated Hospital of Xi’an Jiaotong University, Xi’an, 710061 Shaanxi China; 4 Department of Nurse, Shaanxi University of Chinese Medicine, Xianyang, 712046 Shaanxi China; 5 School of Nurse, Xi’an Jiaotong University, Xi’an, 710061 Shaanxi China; 6 Department of Infectious Diseases, The First Affiliated Hospital of Xi’an Jiaotong University, No. 277 Yanta West Road, Xi’an, 710061 Shaanxi China

**Retraction Note to: Cancer Cell International (2020) 20:216**

10.1186/s12935-020-01307-7

The Editor in Chief has retracted this article because after publication it was noted that Fig. 6C has been previously published in another publication [1] and due to an overlap of the image in Fig. 2D with another publication [2]. The Editor in Chief has therefore lost confidence in the integrity of the results of this article.

Nan Yang has agreed to this retraction on behalf of all authors.
